# Putative Protein Discovery from Microalgal Genomes as a Synthetic Biology Protein Library for Heavy Metal Bio-Removal

**DOI:** 10.3390/biology11081226

**Published:** 2022-08-17

**Authors:** Toungporn Uttarotai, Nilita Mukjang, Natcha Chaisoung, Wasu Pathom-Aree, Jeeraporn Pekkoh, Chayakorn Pumas, Pachara Sattayawat

**Affiliations:** 1 Department of Biology, Faculty of Science, Chiang Mai University, Chiang Mai 50200, Thailand; 2 Department of Entomology and Plant Pathology, Faculty of Agriculture, Chiang Mai University, Chiang Mai 50200, Thailand; 3 Research Center in Bioresources for Agriculture, Industry and Medicine, Chiang Mai University, Chiang Mai 50200, Thailand; 4 Research Center of Microbial Diversity and Sustainable Utilization, Faculty of Science, Chiang Mai University, Chiang Mai 50200, Thailand

**Keywords:** synthetic biology, microalgae, bio-removal, wastewater treatment, alphafold

## Abstract

**Simple Summary:**

Nowadays, heavy metal polluted wastewater is one of the global challenges that leads to an insufficient supply of clean water. Taking advantage of what nature has to offer, several organisms, including microalgae, can natively bioremediate these heavy metals. However, the effectiveness of such processes does not meet expectations, especially with the increasing amount of pollution in today’s world. Therefore, with the goal of creating effective strains, synthetic biology via bioengineering is widely used as a strategy to enhance the heavy metal bio-removing capability, either by directly engineering the native ability of organisms or by transferring the ability to a more suitable host. In order to do so, a list of genes or proteins involved in the processes is crucial for stepwise engineering. Yet, a large amount of information remains to be discovered. In this work, a comprehensive library of putative proteins that are involved in heavy metal bio-removal from microalgae was constructed. Moreover, with the development of machine learning, the 3D structures of these proteins are also predicted, using machine learning-based methods, to aid the use of synthetic biology further.

**Abstract:**

Synthetic biology is a principle that aims to create new biological systems with particular functions or to redesign the existing ones through bioengineering. Therefore, this principle is often utilized as a tool to put the knowledge learned to practical use in actual fields. However, there is still a great deal of information remaining to be found, and this limits the possible utilization of synthetic biology, particularly on the topic that is the focus of the present work—heavy metal bio-removal. In this work, we aim to construct a comprehensive library of putative proteins that might support heavy metal bio-removal. Hypothetical proteins were discovered from *Chlorella* and *Scenedesmus* genomes and extensively annotated. The protein structures of these putative proteins were also modeled through Alphafold2. Although a portion of this workflow has previously been demonstrated to annotate hypothetical proteins from whole genome sequences, the adaptation of such steps is yet to be done for library construction purposes. We also demonstrated further downstream steps that allow a more accurate function prediction of the hypothetical proteins by subjecting the models generated to structure-based annotation. In conclusion, a total of 72 newly discovered putative proteins were annotated with ready-to-use predicted structures available for further investigation.

## 1. Introduction

**Figure 1:**
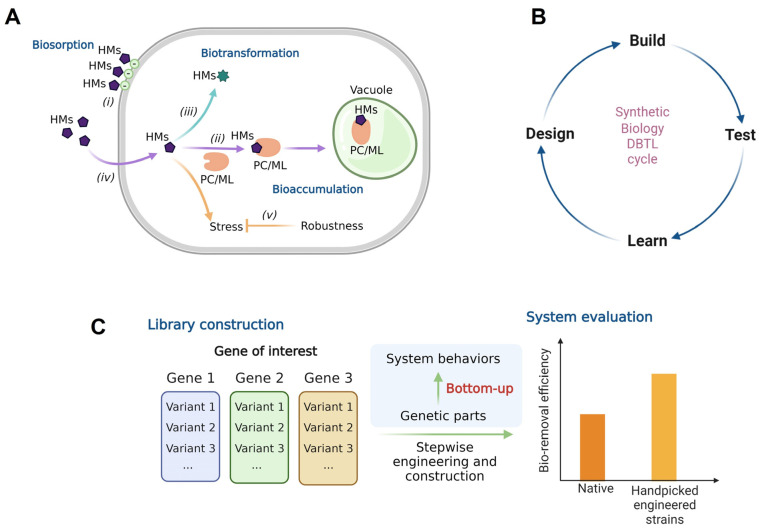
Microalgal heavy metal bio-removing mechanisms and the use of synthetic biology for system enhancement. (**A**) Mechanisms and cellular functions used by microalgae to bio-remove heavy metals (HMs) from wastewater, adapted from Sattayawat et al., 2021 [7]; (*i*) biosorption, (*ii*) bioaccumulation (*iii*) biotransformation and (*iv*) cellular uptake of heavy metals via transporters (*v*) cellular responses to heavy metal-induced stresses. (**B**) Synthetic biology Design-Build-Test-Learn (DBTL) cycle. (**C**) Genetic part library construction to facilitate synthetic biology-based approaches for heavy metal bio-removal enhancement. The figure was created using https://biorender.com (accessed on 5 July 2022).

Heavy metal contaminated wastewater has been a major global concern that directly affects the human population [1,2]. A considerable amount of heavy metals is released into the environment by several industries. The steel industry, for example, is a well-known source of heavy metal contamination [3]. Not only do industrial processes cause pollution, but the products from many industries, such as batteries, are also a major source of contamination [4,5]. Microalgae are promising bio-removers of contaminated heavy metals from wastewater effluents [6]. In our latest review, we summarized three mechanisms used by microalgae to bio-remove heavy metals from wastewater: biosorption, bioaccumulation and biotransformation [7]. In brief, biosorption is a process by which microalgae absorb heavy metal ions onto their cell surface; the anionic composition plays a role in attracting positive heavy metal ions (Figure 1A). This allows the cells to remove heavy metals from aqueous phases by simply attaching them to the cell surface. Interestingly, this process does not need the cells to be in an active form, meaning that dead cells can also function similarly to the living cells as long as the heavy metals can attach to the cell surface. Bioaccumulation is another process by which heavy metals are taken up by microalgal cells and later sequestered in vacuoles or specialized compartments. Lastly, biotransformation allows the conversion of toxic heavy metals to lesser or non-toxic forms using intracellular enzymes. Living cells are required for the latter two mechanisms to work continuously. Moreover, other native cellular functions, such as metal transportation and cellular responses to stress, could also impact the cell ability to bio-remove heavy metals. Heavy metal transporters also play a role in these processes as they determine the efficiency of heavy metal uptake for bioaccumulation and biotransformation. To put it simply, the more heavy metals are taken up, the greater is the possibility that they can be accumulated and transformed. Moreover, cellular responses to heavy metal-induced stresses have been reported to improve cells’ ability to remove heavy metals [8,9]. This is because the cell itself is able to tolerate higher levels of heavy metals while continuing to function at a relatively higher concentration of heavy metals. 

Several microalgae have been reported to bio-remove heavy metals through the aforementioned mechanisms [6]. *Chlorella* and *Scenedesmus* are two groups of microalgae exhibiting their potential as heavy metal bio-removers. *Chlorella* has been well-known for its ability as a heavy metal biosorbent and, in one study, could effectively remove 220 mg of Cu^+2^ per one gram of cells [10]. Similarly, *Scenedesmus* is another group of microalgae that has been practically used for wastewater treatment in several parts of the world [11], though its application may have not been as extensive; a bio-removal capability of 574 mg/g of cells was reported from this group of microalgae [12,13]. Moreover, other microalgae such as *Tetraselmis,* *Porphyridium,* and *Pseudochlorococcum* have been reported to effectively bio-remove heavy metals [6,14,15], but since they are relatively underexplored, only one genome sequence of *Tetraselmis* and *Porphyridium* was reported for each genus, and none was reported for *Pseudochlorococcum* in the NCBI database (www.ncbi.nlm.nih.gov, accessed on 20 June 2022). As the aim of this study is to construct a comprehensive library of proteins based on reported genome sequences, *Chlorella* and *Scenedesmus* were selected, given the relatively high number of their available genome sequences. 

Even though these processes in microalgae may sound as though they hold great promise, there are still limitations in the bio-removing capabilities of microalgae [16]. Thus, there is potential for improvement, particularly in the development of a supreme microalgal chassis for heavy metal contaminated wastewater treatment. Synthetic biology is currently one of the most rapidly growing biological disciplines, with the aim of using integrated knowledge of biology and bioengineering to develop or redesign biological systems with specific functionalities. To provide a related example, an engineered *Chlorella* sp. DT was constructed by expressing a heterologous mercuric reductase from *Bacillus megaterium* MB1 and showed 2-fold increase in mercury bio-removal compared with the wild type [17]. Such techniques allow us to design novel biological systems as solutions to tackle unsolved challenges. 

We propose that microalgal native abilities to bio-remove heavy metals could be enhanced via synthetic biology-based approaches with the proposed strategies previously listed [7]. Generally, the study of synthetic biology follows a guideline called “Design-Build-Test-Learn” or DBTL cycle (Figure 1B), which allows systematic implementation of the new system. Interestingly, the work on synthetic biology to enhance heavy metal bio-removal is not well-established compared with other microorganisms (i.e., *Escherichia coli* and cyanobacteria), and indeed this is true of microalgae in general. This may be related to the low availability of genetic tools and techniques for microalgae as synthetic biology builds on genetic manipulation. More importantly, synthetic biology often applies bottom-up approaches to facilitate the design, in which genetic parts are handpicked and put together to construct novel systems. In this case, the availability of genetic parts is crucial for stepwise implementation. In addition to regulatory genetics (e.g., promoter, ribosome binding site, etc.), a library of genes responsible for significant properties of the systems is also important. To illustrate this, Figure 1C depicts the simplified workflow of a bottom-up approach for synthetic biology and the importance of genetic part libraries.

As the genetic part library—a collection of genetic parts and related genes—is essential for structured design, genes involved in bio-removal should be identified to improve microalgal abilities or even to employ such abilities in other chassis. Previously, a unicellular microalga, *Chlamydomonas*, has been genetically engineered to enhance its abilities as a heavy metal bio-remover. As a result, the engineered strains showed significantly improved bio-removing capability [8,9,18,19,20]. Certainly, this demonstrates the potential of employing synthetic biology to enhance the cell’s native ability for heavy metal bio-removal. However, on the basis of extremely limited data, systematic investigation is yet to be achieved, which emphasizes the importance of this work. Thus, we aim to construct a library of potential proteins involved in heavy metal bio-removal for further use via synthetic biology. 

With a rapid growth of bioinformatics, a number of tools have been developed to facilitate the analysis of biological data. Basic Local Alignment Search Tool or BLAST from National Center for Biotechnology Information (NCBI) is one of the pioneer tools with a great number of users up to today. Many web interfaces designed for function prediction of the proteins from amino acid sequences and/or protein structures were also developed to allow stepwise prediction of the proteins. Pfam, InterProScan, SUPERFAMILY 2.0, and CATH are among the commonly use online tools [21,22]. Not only were the tools for functional annotation developed, well-known web servers such as SignalP were also developed to predict whether the proteins contain signal peptides or not [23]. Function prediction of hypothetical proteins from whole genome sequences has been demonstrated previously. One early example is the use of the aforementioned tools to annotate the function of all hypothetical proteins from *Haemophilus influenzae* Rd KW20 genome aiming to use the information retrieved for medical applications [22]. Later, in another work, hypothetical proteins from a Gram-negative bacterium, *Litorilituus sediminis*, were computationally predicted to be tumor-suppressors [21]. Apart from the medical point of view, hypothetical proteins that play a role in cold adaptation in *Pseudomonas* sp. were characterized using the similar computational workflow. However, all of these works follow the same general pattern in that they aim to annotate and characterize hypothetical proteins from a single organism, and/or only proteins involved in specific functions were targeted. This is in contrast with the present study as we aim to construct a library of putative proteins from whole groups of microalgae and to annotate as many proteins that contribute to heavy metal bio-removal as possible using our proposed approach. Moreover, the development of Alphafold [24], an accurate computational tool for protein folding prediction, caused attention to shift from traditional methods to machine learning to study protein structure. Therefore, to make the information ready to use, in this work, Alphafold2 was also used to predict the 3-dimensional (3D) structures of all putative proteins, and the structures were deposited in a protein structure database for future applications. In summary, a comprehensive library of putative proteins from microalgal genomes was constructed to allow stepwise engineering approaches to enhance heavy metal bio-removal in microalgae and potentially in other organisms. 

## 2. Materials and Methods

**Figure 2:**
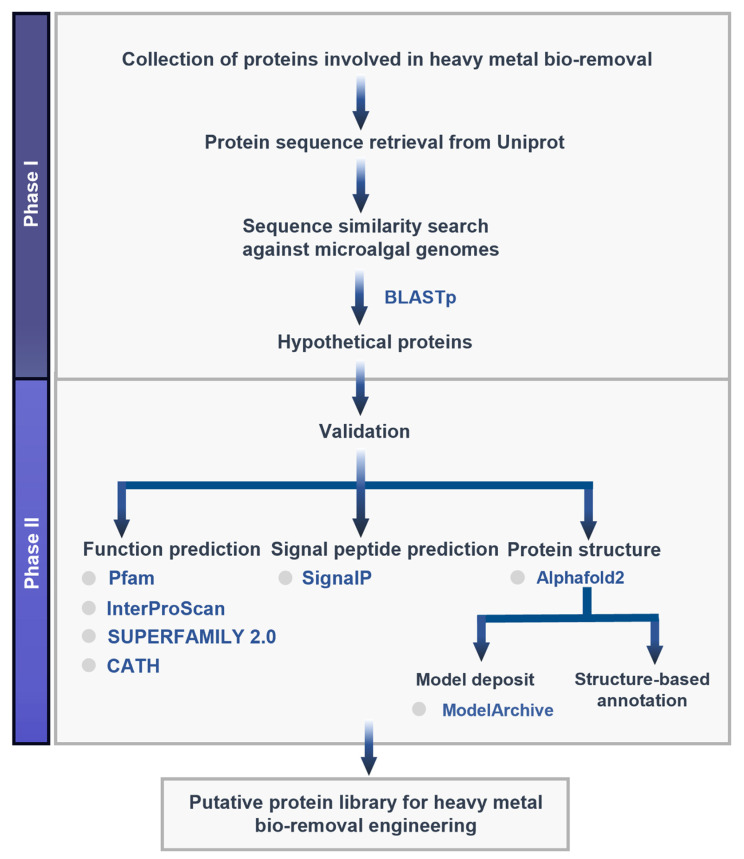
A schematic computational workflow to construct a genetic part library for heavy metal bio-removal. Phase I focuses on protein sequence retrieval and sequence similarity search for protein homologs against available microalgal genomes, whereas Phase II focuses on functional annotation and protein structure modeling to validate their functions.

A simplified workflow and computational online tools used in this study are outlined in Figure 2. The URLs of databases and tools are listed in Table S1. 

To validate the annotation, three positive and negative controls each were annotated along with the putative proteins (Phase II, Figure 2) [25]. These controls were selected based on their experimental validation. The positive controls were validated proteins from microalgae that are involved in heavy metal bio-removal: natural resistance-associated macrophage protein (accession no. XP_001691702.1), ascorbate peroxidase (accession no. AAY26385.1), and glutathione peroxidase (accession no. AFI55004.1), while the negative controls are general characterized proteins that function in cellular processes: elongation factor EF-2 (accession no. NP_001321033.1), transcriptional regulator (accession no. WP_097343503.1), and cytochrome C peroxidase (accession no. WP_016160016.1).

### 2.1. Target Protein Identification and Sequence Retrieval

The terms given in Table S2 were used as inputs to search for proteins in UniprotKB database. The filter “reviewed” is also applied to obtain only the proteins that had been characterized or computationally curated. All search hits were manually confirmed that all proteins selected from this stage possess the expected function, as the search hits may also result in different proteins with the same gene/protein abbreviation. All selected sequences were exported as template sequences for the next step in a FASTA format. 

### 2.2. Sequence Similarity Search

NCBI BLASTp function was used for sequence similarity search, using the obtained sequences as templates against the genomes of two microalgal groups namely *Chlorella* and *Scenedesmus* (Table 1). Non-redundant protein sequences (nr) database and blastp (protein-protein BLAST) algorithm were selected. Once the search results from BLASTp showed hits for each group of proteins, only hypothetical protein sequences with an E-value of less than 1 × 10^−10^ were selected. When searching a database, the E-value is a parameter that indicates how many hits are likely to occur by chance: the lower the E-value, the more significant the match is. It is recommended that the E-value between 1 × 10^−10^–1 × 10^−50^ should, at least, allow a domain match. The E-value between 1 × 10^−50^–1 × 10^−100^ indicates almost identical sequences, and if the E-value is less than 1 × 10^−100^, it suggests identical sequences as described in the Qiagen handbook [26]. However, it is important to note that E-value cutoffs should be considered on a case-by-case basis as they are dependent on the length of the sequence and the size of the databases.

### 2.3. Function Prediction

To primarily predict the functions of the retrieved protein sequences, four different webservers, namely, Pfam, InterProScan, SUPERFAMILY 2.0, CATH, were selected (Table S1). All webservers were previously used to annotate hypothetical proteins from whole genome sequences with a comparative assessment of each tool [29]. 

### 2.4. Signal Peptide Prediction

Signal peptide prediction is used to confirm the primary annotation as the target proteins in this work localize specifically, either as transporters, which localize on the cell membrane, or enzymes that function intracellularly. SignalP is one of the most used webservers for signal peptide identification. This server has recently released its latest version SignalP 6.0 early this year and claimed its ability to detect all types of signal peptides [30]. 

### 2.5. Structure Modeling

Alphafold2 is used for structure prediction in this study. Alphafold is a recent innovative tool to accurately model the protein structures based on the provided protein sequences [24]. With an increased interest in Alphafold, a recent work aiming to make the structure modeling available to all has recently been published [31] and the online tool is free to use under the name ColabFold. In this study, a related version of Alphafold via Google Colab notebook was used (Table S1). The analysis was performed with default parameters and Amber-Relax applied. The genetic database, mmseqs2, was selected. No custom MSA was uploaded to any runs. The filter option was left unchanged since the raw hypothetical protein was run without any trims. The Alphafold was run using pLDDT metric with 512:1024 max msa. Five models were run for each template with the use of ptm to fine-tune the model parameters. Each model was fed back to the neutral network three times. All generated protein structures were deposited in ModelArchive with a list of unique DOI as shown in Table S4.

### 2.6. Structure-Based Functional Annotation 

All protein structures were investigated further to validate the primary annotation in the previous step. ProFunc was used for this purpose. This tool was designed to annotate the proteins based on sequence and structure inputs [32]. In our case, as mentioned, the structures generated from Alphafold2 were used as the template. Option “reverse templates” was examined and the E-value of at least 1 × 10^−6^ was considered, according to the server.

## 3. Results

### 3.1. Target Protein Identification

Microalgae perform three main mechanisms that allow them to bio-remove heavy metals from wastewater effluents. Previously, we have compiled a list of genes with reported use for genetic engineering to enhance microalgal bio-removing capacity [7]. Thus, in this work, we utilize the list as a primary source for protein targets. As part of our effort to investigate all relevant aspects toward heavy metal bio-removal, we have broadened the scope of the search by including more proteins from the literature. A total of 26 protein targets are shown in Table S2, listed separately based on their function in each mechanism. As for bioaccumulation, phytochelatins play an important role in binding with heavy metals, and enzymes involved in the synthesis of phytochelatins namely glutamate cysteine ligase, phytochelatin synthase, and glutathione synthetase [33] were therefore targeted. Although another type of heavy metal binding proteins, metallothioniens, also play a role in the same mechanism, they are directly transcribed and translated from nucleotide sequences as cysteine-rich short peptides [33], which are difficult to search against the genomes. Therefore, this type of heavy metal binding protein is not included in this study. Reductases are a major group of enzymes that detoxify heavy metals by converting them into their less or non-toxic derivatives [34,35]. In our list, three reductases specific to mercury, chromium, and arsenic were used to search against the microalgal genomes, as these enzymes have been reported to alleviate the toxicity of heavy metals in microalgae [18,36]. As previously described, enhanced metal transportation is another strategy that allows better performance of bio-removal, especially to facilitate the bioaccumulation and biotransformation in which enzymes function intracellularly. Several types of transporters have been reported to be responsible for microalgal metal transportation [37]. In this work, we selected a few representatives of heavy metal transporters as our templates. Similarly, five different enzymes were selected as representatives of cellular stress responses. All of these enzymes were reported to take part in cellular responses to heavy metal toxicity in microalgae [38]. As mentioned, biosorption is a mechanism by which heavy metal ions are attracted onto the cell surface of microalgae and, in turn, removed upon removal of the microalgae. However, this mechanism is not a direct effect from proteins synthesized by the cells, but rather indirectly from the composition of the cell surface. To enhance biosorption efficiency, the strategy may include cell surface modification to make the surface components more ionic and attract more heavy metal ions. Such modifications could be achieved by, for example, addition of CXXEE motif onto the cell surface components [39] or inducing biofilm formation [40]. Therefore, this mechanism is not included in our list. The proteins and keywords used are listed in Table S2. However, it should be noted that when using UniprotKB database or other keyword-based tools, the results are not exclusive for some keywords as different meanings can be inferred. To state the obvious, the keyword “inorganic phosphate transporter”, which is a family name of the protein transporters involved in import-export systems of phosphate [41], resulted in a total of 712 hits from UniprotKB search. However, manual selection only showed 52 hits of the inorganic phosphate transporters, the rest of the hit results showed other related proteins such as vacuolar transporter chaperones, glucose-6-phosphate exchangers, and other ATP-binding cassettes. 

**Figure 3:**
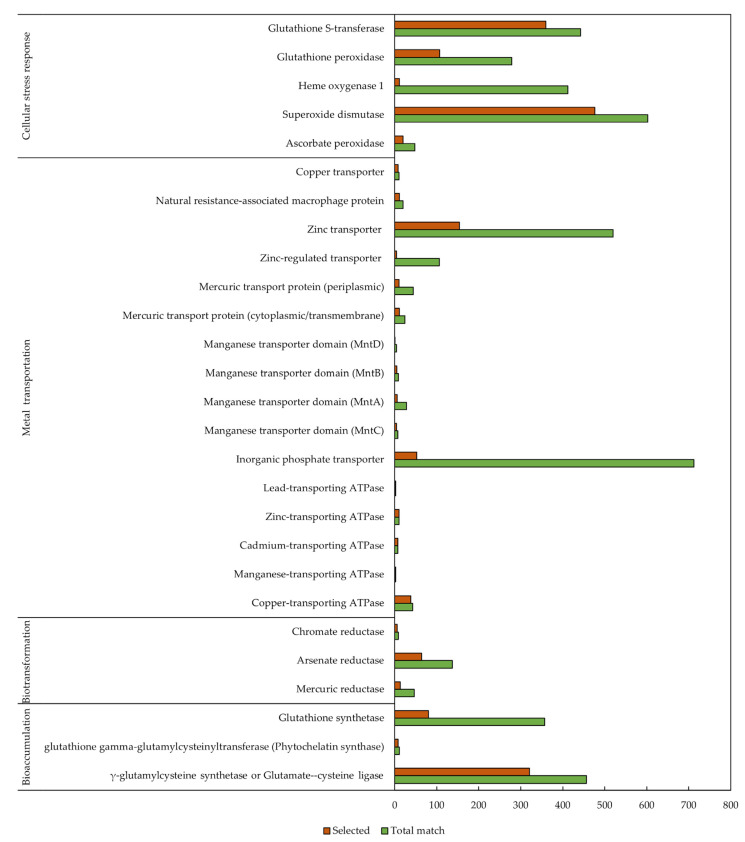
Search results for each group of proteins presented separately by their fundamental roles in heavy metal bio-removing mechanisms. The green bars represent a total number of hits when each keyword was used to search against UniprotKB database, and the orange bars represent the number of hits after manual selection of the proteins.

According to the UniprotKB database, we collectively report the number of hits for each group of proteins (bioaccumulation, biotransformation, heavy metal transporters, and cellular stress responses) as shown in Figure 3. 

### 3.2. Sequence Similarity Search

The sequences of all 27 groups of protein targets were obtained and used as templates for BLASTp against two microalgal genomes, *Chlorella* (taxid 3071) and *Scenedesmus* (taxid 3087). Hypothetical proteins with an E-value lower than 1 × 10^−10^ were obtained. The number of matches is shown in Table 2.

### 3.3. Protein Function Prediction 

The NCBI accession numbers for all protein matches from BLASTp are listed in Table 3. Function and signal peptide prediction for each hypothetical protein were investigated. As we aim to only annotate the uncharacterized proteins, only sequences described as hypothetical proteins were selected and run through function prediction servers as described above. The results for all hypothetical proteins are listed in Table S3. Each bioinformatic server has its own strength and weakness. Thus, using multiple servers would result in a more accurate prediction. A recent publication has compared several webservers for protein prediction and the results revealed that Pfam and InterPro showed the highest scores among the compared webservers [29]. Pfam is a widely used protein family database and tool that is still active with a recent update in 2021 [42]. InterProScan is another well-known functional classification tool that is based on several databases [43]. SUPERFAMILY, another webserver used in this study, was also considered the second-best server according to the aforementioned study. This tool was designed to predict both superfamily and family of the protein sequences [44]. In the same comparative report [29], SBase was also used and demonstrated to have high overall scores for accuracy, sensitivity, specificity, and ROC analysis, yet the server was last updated in 2006; therefore, SBase was not included in the present study. Lastly, although CATH did not score much in the aforementioned comparison especially regarding the specificity, according to our predictions, CATH produced relatively specific results (Table S3). To provide an explanation, when CATH was used to predict the function of a template (accession no. XP_005845237.1) expected to be a manganese transporter, CATH predicted the protein as “ABC transporter G family member 22”, whilst Pfam predicted as “ABC transporter”. Moreover, CATH is the only webtool that allows a specific annotation of copper-transporting ATPase (Table S3). Pfam, on the other hand, predicted the function of ATPases separately for different protein domains. For example, copper-transporting ATPase shows 4 predicted functions: (1) cation transporting ATPase, C-terminus, (2) E1-E2 ATPase, (3) haloacid dehalogenase-like hydrolase (CL0137), and (4) cation transporter/ATPase, N-terminus, which are basic structures for ATPase activity [45]. Similarly, InterProScan also resulted in a list of molecular functions that could be inferred as ATPase rather than stating that the proteins are ATPase (Table S3). 

Signal peptide prediction was used to double-validate the predicted function of the hypothetical proteins. SignalP is a well-reputed webserver for signal peptide prediction. A report on the comparison of signal peptide prediction suggests that the use of SignalP 4.1 is most consistent compared with the other versions [46]. However, the most recent version, SignalP 6.0, was released after that [30]. Interestingly, the results showed that most of the proteins did not contain signal peptides, even though some of them were expected to be transporters (Table 3).

### 3.4. Homology Modeling

**Figure 4:**
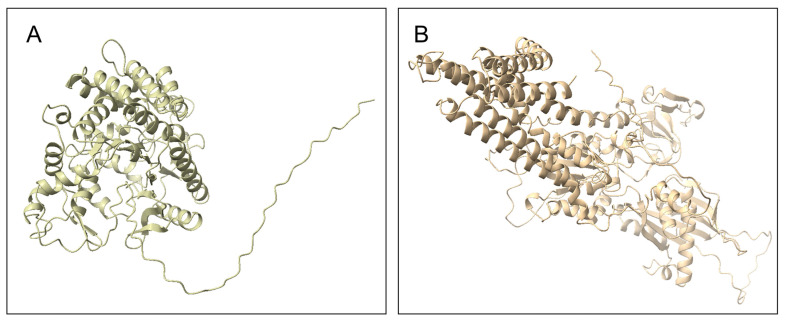
Protein structures predicted using Alphafold2. (**A**) Glutamate-cysteine ligase (accession no. KAG7671258.1) and (**B**) Copper-transporting ATPase (accession no. XP_005845243.1). The structures are visualized by UCSF ChimeraX [48].

All hypothetical protein sequences primarily characterized to have the same function and signal peptide as their templates were modeled using Alphafold2. Amber-relax was applied to generate more accurate models. Examples of protein structures generated are presented in Figure 4. It should be noted that a hypothetical protein from *Scenedesmus* sp. PABB004 (accession no. KAF8061310.1) is 1553 amino acid long; therefore, this protein was not modeled through Alphafold2 as the recommended longest sequence was 1400 amino acids. When using Alphafold, the confidence measure of the models can be evaluated using a pLDDT score (0–100), by which the guidance is as follows; regions with a pLDDT score of more than 90 are considered highly accurate. Regions with a pLDDT score between 70 and 90 are considered generally good and regions with a pLDDT score between 50 and 70 are considered with low confidence. In our case, out of 72 models, 31 models showed the average pLDDT scores of more than 90, 31 models showed the average scores between 70 and 90, and 10 models showed the average scores between 50 and 70 (Table S4). All generated models were deposited in ModelArchive with the accession ID listed in Table S4. It is also important to note that the commonly used Protein Data Bank (PDB) currently only accepts experimental model depositions [47].

### 3.5. Structure-Based Annotation

Using the created models as a resource, ProFunc—a structure-based functional annotation—was used to annotate the proteins with structure inputs. ProFunc revealed that the majority of the annotated proteins possess the same functions as primarily annotated in Table 3. However, 7 structures out of 72 protein structures were annotated with different functions as shown in Table S5. To elaborate, two structures with manganese transporter domain (MntA) putative functions were annotated as ATP-bound human transporter found in retina and human sterol transporter, respectively. Two zinc-regulated/zinc transporters were annotated as NH_3_ transporter from *Nitrosomonas europaea* and multidrug transporter from *Lactobacillus lactis*. One ascorbate peroxidase was annotated as cytochrome C peroxidase and two glutathione *S*-transferases were annotated as apo-dehydroascorbate reductase and glutathione-bound dehydroascorbate reductase, respectively.

## 4. Discussion

The rise in the number of whole genome sequences prompts a rapid development of computational tools for the analysis of this available data. A webtool was previously developed to help predict the functions of genes from microalgal genomes and is currently active via http://pathways.mcdb.ucla.edu/algal/index.html (accessed on 23 May 2022); however, the database’s scope is limited only to two microalgae, *Chlamydomonas reinhardtii* and *Chlorella* NC64A. Thus, other means of methods are still useful, particularly if the genes or proteins from other microalgal strains are in question. 

In our work, it is obvious that BLASTp resulted in greater numbers of hits against *Chlorella* than *Scenedesmus*. The most straightforward explanation for this is because the number of reported whole genome sequences of *Chlorella* is significantly higher (21 genomes; www.ncbi.nlm.nih.gov, accessed on 23 May 2022), in comparison with that of *Scenedesmus* (6 genomes; accessed on 23 May 2022). Moreover, *Chlorella* is considered a frequently used microalga for wastewater treatment, despite the increased attention toward *Scenedesmus*. Noticeably, no enzymes implicated in the bioaccumulation mechanism were found from *Scenedesmus* genomes. This could be due to the explanation stated above, or it may suggest that the *Scenedesmus* does not actually use this mechanism. However, it should also be emphasized that glutathione peroxidase, which is a common enzyme found in most organisms to protect the cell from oxidative damage [49], was also not found in *Scenedesmus*. This may suggest that the use of BLASTp to retrieve proteins from microalgal genomes could be further improved. 

Available webtools for functional annotation are useful, especially to primarily screen for protein sequences with particular functions. Interestingly, protein sequences retrieved when using reductase enzymes as templates were not explicitly identified as reductases. Although two proteins (accession no. KAG7668560.1 and XP_005845177.1) were identified by CATH as thioredoxin reductase, which is often seen coupled with arsenate reductases [50,51], the other servers identified otherwise. Moreover, it is undeniably challenging to predict the functions of transporters as they consist of several domains to form a functional protein [37,52] and they are often broadly specific toward different substrates [53]. This was illustrated clearly from BLASTp search where all cadmium-, zinc- and lead-transporting ATPase showed the same hit results with copper-transporting ATPase (Table S3), suggesting that their structures are similar, and they are broadly specific to several heavy metals. All heavy metal transporting ATPases are classified as P-type ATPases and contain (1) E1-E2 ATPase (2) hydrolase (3) cation ATPase domains [54]. In this regard, Pfam and InterProScan broadly annotate the molecular functions of different regions on the hypothetical proteins as ATP hydrolysis activity, ATP binding, transporter activity, nucleotide binding (Table S5). Though this fits the definition of P-ATPase, it suggests that the specificity of these tools could be further improved. We suggest that the use of these servers is still applicable, but further interpretation is required. Noticeably, the results from SUPERFAMILY 2.0 and CATH showed several calcium-transporting ATPase hits for all heavy metal transporting ATPase, which is because heavy metal transporting ATPase could also function as a Ca^2+^ pump [55]. 

Alphafold is a breakthrough computational method for protein structure prediction with atomic level accuracy. Using coding-based platform, this makes it challenging for researchers outside of the computational fields to apply Alphafold to their studies. Recently, a few reports, including from the Alphafold creators themselves, have developed relatively more user-friendly Alphafold on Google Colab platform [31]. With such widely accessible platforms, the number of models generated from Alphafold is anticipated to sharply increase in the near future. In our work, we observed that a majority of the predicted protein structures showed long amino acid chains that do not form secondary structures with the rest of the protein region (Figure 4), which could be because the actual start points of the protein sequences retrieved from BLASTp were not accurately identified. This instantly brought us back to the signal peptide prediction from SignalP server, as this tool only allows the prediction of the first 70 amino acids from the N-terminus, which means that if the hypothetical protein sequences retrieved from NCBI contain more than 70 amino acids upstream of the actual start point, the prediction using SignalP would not be accurate.

Though Alphafold has its own protein database (https://alphafold.ebi.ac.uk/ (accessed on 20 June 2022)), the submission is not made available. Therefore, in this study, we submitted our Alphafold models to ModelArchive, which is the only database that allows submission of modeling-based protein structures. 

Structure-based annotation is considered a more accurate method for predicting protein function than homology-based prediction, as the latter often considers only the sequence similarity of the input proteins and their homologs, while the former also considers other factors, including protein binding pocket [56]. In this work, ProFunc is used as a method to validate the protein prediction from primary screening. The results were not surprising, as most of the proteins were annotated to have the same function as from homology-based prediction. This assures that the homology-based annotation could be used to some extent. Interestingly, when looking at seven proteins with different annotation results, it was noticed that the transporters were also annotated as transporters but with specified substrates/ligands. It is important to point out that since homology-based annotation only allows a broad annotation of transporters as unspecified transporters, structure-based annotation allows the substrates/ligands of the transporters to also be identified. Unfortunately, in these cases, the substrates identified were not heavy metals as expected. In the case of enzymes, however, predicted ascorbate peroxidase from homology-based prediction was predicted as cytochrome C peroxidase in this step. This could be linked to their highly similar protein structures, even though they have different substrate-binding sites [57]. Therefore, this observation demonstrates that structure-based annotation allows a more accurate prediction of the protein function, especially when the proteins have similar structures. Two structures of glutathione S-transferases were annotated as apo-dehydroascorbate reductase and glutathione-bound dehydroascorbate reductase. The dehydroascorbate reductase (DHRA) is a member of glutathione *S*-transferase superfamily [58] suggesting that ProFunc could identify subgroups of the proteins in some cases. Moreover, the challenge encountered when using Pfam and InterProscan in the primary annotation was not presented when using ProFunc. To give an example, instead of predicting a heavy metal transporting ATPase (accession no. XP_005851032.1) separately for each region as stated above, ProFunc predicted the protein to be similar to zinc-transporting P_IB_-type ATPase. Overall, the use of structure-based annotation is a useful approach that accurately annotates the protein sequences, especially in our case, when investigating the proteins with similar structures. Furthermore, it allows the annotation of transporters as a whole protein rather than separated domains. However, it should also be taken into account that out of all 72 protein structures used as templates for structure-based annotation via ProFunc, only 31 of them were considered highly accurate predictions according to the Alphafold2 confidence measures.

To validate the proposed approach, natural resistance-associated macrophage protein, ascorbate peroxidase, and glutathione peroxidase with experimentally confirmed activities were used as positive controls. The results showed that after extensive annotation following our workflow, the protein sequences were annotated as expected. Experimentally validated elongation factor EF-2, transcriptional regulator, and cytochrome C peroxidase were selected as negative controls. These proteins function in general cellular processes, and in the case of cytochrome C peroxidase, it has a very similar structure with ascorbate peroxidase and is often shown as matches when ascorbate peroxidase is blasted. Therefore, this choice of negative control would allow the assessment of our approach even when influenced by these factors. The results showed that these proteins were annotated to possess their validated functions, which demonstrates that the workflow presented here could distinguish between the two very similar-structured proteins.

Ultimately, the applications of our protein library could range from selection of the proteins from our library for a single engineering design to combinatorial optimization of all genetic parts. At present, the construction of synthetic biology-based systems mostly relies on previously reported genes or proteins, which limits the possibility of the design and the optimization. For example, engineering of a microalga to enhance heavy metal bioremediation was recently reported and the work utilized gene sequences from *Arabidopsis* that were experimentally characterized prior to the study [18]. This limits the selection of the genes to only the reported ones and raises the question whether the reported genes are the best homologs that there are. Therefore, selection of the genes from our library could provide more choices and allow a stepwise design [59]. Moreover, the predicted protein structures from our work could be used for further computational analysis to predict the activity of the proteins and compare homologs prior to the actual engineering step. Molecular docking is a recent field of protein study that investigates the compatibility of proteins and their ligands or substrates, which can be used to predict the activity of the proteins. To provide a related example, a recent work performed molecular docking to confirm the binding between chromium ion and cell surface proteins in biosorption mechanism [60]. 

## 5. Conclusions

In this work, we aim to construct a comprehensive library of putative proteins from *Chlorella* and *Scenedemus* genomes to facilitate synthetic biology-based engineering for heavy metal bio-removal. We selected 27 different groups of protein targets based on their reported capability as proteins involved in heavy metal bio-removing mechanisms. We found a total of 72 putative proteins. Among these, 65 were exclusively annotated to possess the same functions as their templates. The protein structures of all annotated proteins were also generated and deposited in the protein structure archive for any further use. This study, therefore, provides a putative protein library that could be used as a database for synthetic biologists to handpick the proteins for engineering purposes with readily available structures for additional investigations. Nonetheless, it should be kept in mind that some limitations remain when using our approach for putative protein discovery. First, as encountered in our case, the relatively low number of reported microalgal genomes limits the number of discoverable proteins. Second, the current platform of Alphafold2 only allows up to 1400 amino acid long proteins as templates for modeling. This restriction is another factor to be considered if large proteins are in question. If these limitations are mitigated, a larger number of putative proteins are anticipated to be discovered and annotated using our approach. 

## Figures and Tables

**Table 1:** Microalgae with reported HM bio-removal capability.

Microalga	Reported Mechanism	Reference
*Chlorella* (taxid 3071)	Biosorption, bioaccumulation, biotransformation	[27]
*Scenedesmus* (taxid 3087)	Bioaccumulation, biotransformation, cellular stress response	[28]

**Table 2:** Number of hypothetical proteins obtained from BLASTp analysis.

Protein	Microalgal Genome	Hypothetical Protein	Hypothetical Protein after Primary Function Prediction
Bioaccumulation			
*γ*-Glutamylcysteine synthetase or Glutamate—cysteine ligase	*Chlorella*	4	2
	*Scenedesmus*	1	0
Phytochelatin synthase	*Chlorella*	2	2
	*Scenedesmus*	0	0
Glutathione synthetase	*Chlorella*	2	2
	*Scenedesmus*	0	0
Inorganic phosphate transporter	*Chlorella*	10	7
	*Scenedesmus*	0	0
Biotransformation			
Mercuric reductase	*Chlorella*	10	0
	*Scenedesmus*	5	0
Arsenate reductase	*Chlorella*	6	0
	*Scenedesmus*	1	0
Chromate reductase	*Chlorella*	0	0
	*Scenedesmus*	0	0
Metal transportation			
Copper-transporting ATPase	*Chlorella*	26	9
	*Scenedesmus*	8	2
Manganese-transporting ATPase	*Chlorella*	9	0
	*Scenedesmus*	1	0
Cadmium-transporting ATPase	*Chlorella*	22	0
	*Scenedesmus*	4	0
Zinc-transporting ATPase	*Chlorella*	25	0
	*Scenedesmus*	6	0
Lead-transporting ATPase	*Chlorella*	11	0
	*Scenedesmus*	1	0
Manganese transporter domain (MntA)	*Chlorella*	43	1
	*Scenedesmus*	10	1
Manganese transporter domain (MntB)	*Chlorella*	27	1
	*Scenedesmus*	6	1
Manganese transporter domain (MntC)	*Chlorella*	0	0
	*Scenedesmus*	0	0
Manganese transporter domain (MntD)	*Chlorella* *S* *cenedesmus*	0 0	0 0
Mercuric transport protein (cytoplasmic/transmembrane)	*Scenedesmus*	0	0
	*Scenedesmus*	0	0
Mercuric transport protein (periplasmic)	*Chlorella*	0	0
	*Scenedesmus*	0	0
Zinc-regulated transporter (ZRT)	*Chlorella*	5	1
	*Scenedesmus*	1	0
Zinc transporter (ZIP)	*Chlorella*	13	1
	*Scenedesmus*	4	1
Natural resistance-associated macrophage protein	*Chlorella*	2	2
	*Scenedesmus*	0	0
Copper transporter	*Chlorella*	0	0
	*Scenedesmus*	0	0
Cellular tolerance			
Ascorbate peroxidase	*Chlorella*	9	8
	*Scenedesmus*	0	0
Superoxide dismutase	*Chlorella*	10	9
	*Scenedesmus*	4	4
Heme oxygenase	*Chlorella*	5	4
	*Scenedesmus*	2	2
Glutathione peroxidase	*Chlorella*	15	10
	*Scenedesmus*	0	0
Glutathione *S*-transferase	*Chlorella*	40	20
	*Scenedesmus*	11	2

**Table 3:** List of selected hypothetical protein matches when the template sequences were used to BLASTp against the selected microalgal genomes.

Genome	Accession No.	Signal Peptide *	Putative Function
Bioaccumulation			
*Chlorella desiccata* (nom. nud.)	KAG7671258.1	N	Glutamate-cysteine ligase
*Chlorella variabilis*	XP_005844806.1	N	Glutamate-cysteine ligase
*Chlorella desiccata (nom. nud.)*	KAG7668718.1	N	Phytochelatin synthase
*Chlorella variabilis*	XP_005845668.1	N	Phytochelatin synthase
*Chlorella desiccata (nom. nud.)*	KAG7673317.1	N	Glutathione synthetase
*Chlorella variabilis*	XP_005847003.1	N	Glutathione synthetase
Metal transportation			
*Chlorella variabilis*	XP_005845243.1	Y	Heavy metal transporting ATPase
*Chlorella variabilis*	XP_005851032.1	Y	Heavy metal transporting ATPase
*Scenedesmus* sp. NREL 46B-D10	KAF6264708.1	Y	Manganese transporter domain (MntA) **
*Chlorella desiccata* (nom. nud.)	KAG7670010.1	Y	Manganese transporter domain (MntB)
*Chlorella variabilis*	XP_005845281.1	Y	Manganese transporter domain (MntB) **
*Chlorella variabilis*	XP_005844148.1	Y	Zinc-regulated transporter (ZRT)/Zinc transporter (ZIP) **
*Chlorella variabilis*	XP_005846850.1	Y	Zinc-regulated transporter (ZRT)/Zinc transporter (ZIP) **
*Chlorella desiccata* (nom. nud.)	KAG7667456.1	Y	Zinc-regulated transporter (ZRT)/Zinc transporter (ZIP)
*Chlorella desiccata* (nom. nud.)	KAG7675010.1	N	Natural resistance-associated macrophage protein
*Chlorella variabilis*	XP_005847346.1	N	Natural resistance-associated macrophage protein
Cellular tolerance			
*Chlorella variabilis*	XP_005842918.1	N	Ascorbate peroxidase
*Chlorella variabilis*	XP_005847371.1	N	Ascorbate peroxidase
*Chlorella desiccata* (nom. nud.)	KAG7671272.1	N	Ascorbate peroxidase **
*Chlorella desiccata* (nom. nud.)	KAG7672626.1	N	Ascorbate peroxidase
*Chlorella variabilis*	XP_005842951.1	N	Ascorbate peroxidase
*Chlorella desiccata* (nom. nud.)	KAG7671850.1	N	Ascorbate peroxidase
*Chlorella variabilis*	XP_005851196.1	N	Ascorbate peroxidase
*Chlorella desiccata* (nom. nud.)	KAG7671273.1	N	Ascorbate peroxidase
*Chlorella variabilis*	XP_005852313.1	N	Superoxide dismutase
*Chlorella variabilis*	XP_005852314.1	N	Superoxide dismutase
*Chlorella variabilis*	XP_005850331.1	N	Superoxide dismutase
*Chlorella variabilis*	XP_005850533.1	N	Superoxide dismutase
*Chlorella variabilis*	XP_005850825.1	N	Superoxide dismutase
*Chlorella variabilis*	XP_005851580.1	N	Superoxide dismutase
*Chlorella desiccata* (nom. nud.)	KAG7672127.1	N	Superoxide dismutase
*Chlorella desiccata* (nom. nud.)	KAG7672915.1	N	Superoxide dismutase
*Chlorella desiccata* (nom. nud.)	KAG7673432.1	N	Superoxide dismutase
*Scenedesmus* sp. NREL 46B-D3	KAF6253844.1	N	Superoxide dismutase
*Scenedesmus* sp. PABB004	KAF8054759.1	N	Superoxide dismutase
*Scenedesmus* sp. PABB004	KAF8070899.1	N	Superoxide dismutase
*Scenedesmus* sp. PABB004	KAF8072345.1	N	Superoxide dismutase
*Chlorella variabilis*	XP_005851913.1	N	Heme oxygenase 1
*Chlorella desiccata* (nom. nud.)	KAG7671693.1	N	Heme oxygenase 1
*Chlorella variabilis*	XP_005845884.1	N	Heme oxygenase 1
*Chlorella variabilis*	XP_005842792.1	N	Heme oxygenase 1
*Scenedesmus* sp. NREL 46B-D3	KAF6256065.1	N	Heme oxygenase 1
*Scenedesmus* sp. PABB004	KAF8061310.1	N	Heme oxygenase 1
*Chlorella variabilis*	XP_005852198.1	N	Glutathione peroxidase
*Chlorella desiccata* (nom. nud.)	KAG7666639.1	N	Glutathione peroxidase
*Chlorella variabilis*	XP_005847444.1	N	Glutathione peroxidase
*Chlorella variabilis*	XP_005848232.1	N	Glutathione peroxidase
*Chlorella variabilis*	XP_005851691.1	N	Glutathione peroxidase
*Chlorella desiccata* (nom. nud.)	KAG7666823.1	N	Glutathione peroxidase
*Chlorella variabilis*	XP_005850288.1	N	Glutathione peroxidase
*Chlorella desiccata* (nom. nud.)	KAG7675006.1	N	Glutathione peroxidase
*Chlorella variabilis*	XP_005844151.1	N	Glutathione peroxidase
*Chlorella desiccata* (nom. nud.)	KAG7671063.1	N	Glutathione peroxidase
*Chlorella desiccata* (nom. nud.)	KAG7667083.1	N	Glutathione *S*-transferase **
*Chlorella desiccata* (nom. nud.)	KAG7667402.1	N	Glutathione *S*-transferase
*Chlorella desiccata* (nom. nud.)	KAG7667544.1	N	Glutathione *S*-transferase
*Chlorella desiccata* (nom. nud.)	KAG7667774.1	N	Glutathione *S*-transferase
*Chlorella desiccata* (nom. nud.)	KAG7667817.1	N	Glutathione *S*-transferase
*Chlorella desiccata* (nom. nud.)	KAG7669352.1	N	Glutathione *S*-transferase
*Chlorella desiccata* (nom. nud.)	KAG7669598.1	N	Glutathione *S*-transferase **
*Chlorella desiccata* (nom. nud.)	KAG7670514.1	N	Glutathione *S*-transferase
*Chlorella desiccata* (nom. nud.)	KAG7675170.1	N	Glutathione *S*-transferase
*Chlorella variabilis*	XP_005843180.1	N	Glutathione *S*-transferase
*Chlorella variabilis*	XP_005845006.1	N	Glutathione *S*-transferase
*Chlorella variabilis*	XP_005845127.1	N	Glutathione *S*-transferase
*Chlorella variabilis*	XP_005845396.1	N	Glutathione *S*-transferase
*Chlorella variabilis*	XP_005847002.1	N	Glutathione *S*-transferase
*Chlorella variabilis*	XP_005848700.1	N	Glutathione *S*-transferase
*Chlorella variabilis*	XP_005849485.1	N	Glutathione *S*-transferase
*Chlorella variabilis*	XP_005849684.1	N	Glutathione *S*-transferase
*Chlorella variabilis*	XP_005850654.1	N	Glutathione *S*-transferase
*Chlorella variabilis*	XP_005852104.1	N	Glutathione *S*-transferase
*Scenedesmus* sp. NREL 46B-D3	KAF6265595.1	N	Glutathione *S*-transferase

* Note that Y indicates Yes and N indicates No. The cutoff value is 0.1 for Signal Peptide (Sec/SPI). ** Note that these proteins were annotated differently when using ProFunc structure-based annotation.

## Data Availability

All the data associated with this research is included in this article and its supplementary information. Any further information is available upon reasonable request.

## Supplementary Materials

The following supporting information can be downloaded at: https://www.mdpi.com/article/10.3390/biology11081226/s1, Table S1: List of bioinformatic webtools used in this study; Table S2: A list of target proteins in this study and their search results from UniprotKB; Table S3: Function prediction using different webservers; Table S4: Protein structure archive ID; Table S5: Structure-based annotation of the putative proteins.
