# Supplementary material for: Putative Protein Discovery from Microalgal Genomes as a Synthetic Biology Protein Library for Heavy Metal Bio-Removal
Source: Biology (Basel). 2022 Aug 17;11(8):1226. doi: 10.3390/biology11081226 (PMC9405338; doi:10.3390/biology11081226)
Supplement: Supplementary file 1 [file biology-11-01226-s001.zip › Supplementary Information (Table S1).pdf]

Supplementary information

Table S1. List of bioinformatic webtools used in this study

Software	URL	Remark
**Amino acid sequence retrieval**		
Uniprot	https://www.uniprot.org/	Protein database
**Sequence similarity search**		
BLAST: Basic Local Alignment Search Tool	https://www.ncbi.nlm.nih.gov/	BLASTp is used to search for similar sequences from microalgal genomes
**Protein function prediction**		
BLAST: Basic Local Alignment Search Tool	https://www.ncbi.nlm.nih.gov/	Primary function prediction
**Final Validation**		
InterProScan	http://www.ebi.ac.uk/InterProScan/	Function prediction
SUPERFAMILY 2.0	http://supfam.org	Function prediction
CATH	https://www.cathdb.info/	Function prediction
Pfam	http://pfam.xfam.org/	Function prediction
SignalP 5.0	https://services.healthtech.dtu.dk/ service.php?SignalP-5.0	Subcellular localization
Alphafold2	https://github.com/sokrypton/ColabFold	Structure modelling
Modelarchive	https://modelarchive.org/	Structure archive
ProFunc	https://www.hsls.pitt.edu/obrc/index.php?page= URL1125088260	Structure-based annotation
