# Supplementary material for: Putative Protein Discovery from Microalgal Genomes as a Synthetic Biology Protein Library for Heavy Metal Bio-Removal
Source: Biology (Basel). 2022 Aug 17;11(8):1226. doi: 10.3390/biology11081226 (PMC9405338; doi:10.3390/biology11081226)
Supplement: Supplementary file 1 [file biology-11-01226-s001.zip › Supplementary Information (Table S2).pdf]

Table S2. A list of target proteins in this study and their search results from UniprotKB

Protein	Keyword	Number of hits	Number of selected proteins
**Bioaccumulation**			
**γ**-glutamylcysteine synthetase or Glutamate--cysteine ligase	Glutamate--cysteine ligase	456	321
glutathione gamma-glutamylcysteinyltransferase	Phytochelatin synthase	11	8
Glutathione synthetase	Glutathione synthetase	357	80
**Biotransformation**			
Mercuric reductase	mercuric reductase	46	13
Arsenate reductase	arsenate reductase	137	64
Chromate reductase	chrR	9	6
**Metal transportation**			
Heavy metal transporting ATPase (HMA)	Copper-transporting ATPase	43	38
	Manganese-transporting ATPase	2	2
	Cadmium-transporting ATPase	7	7
	Zinc-transporting ATPase	10	10
	Lead-transporting ATPase	2	2
Inorganic phosphate transporter	Inorganic phosphate transporter	712	52
Manganese transporter domain	MntC	7	6
Manganese transporter domain	MntA	28	9
Manganese transporter domain	MntB	9	5

Manganese transporter domain	MntD	4	1
Mercuric transport protein (cytoplasmic/transmembrane)	MerT	24	11
Mercuric transport protein (periplasmic)	MerP	44	10
Zinc-regulated transporter	ZRT	106	4
Zinc transporter	ZIP	520	154
Natural resistance-associated macrophage protein	NRAMP1	20	11
Copper transporter	COPT1	10	8
**Cellular tolerance**			
Ascorbate peroxidase	ascorbate peroxidase	48	20
Superoxide dismutase	superoxide dismutase	602	476
Heme oxygenase	heme oxygenase 1	412	11
Glutathione peroxidase	glutathione peroxidase	279	107
Glutathione S-transferase	glutathione *S*-transferase	442	360

*****Transcriptional factors e.g., metal-tolerant proteins (MTPs), metalloregulatorory proteins are not included in the list
